# A rat model of hypohidrotic ectodermal dysplasia carries a missense mutation in the *Edaradd *gene

**DOI:** 10.1186/1471-2156-12-91

**Published:** 2011-10-21

**Authors:** Takashi Kuramoto, Mayuko Yokoe, Ryoko Hashimoto, Hiroshi Hiai, Tadao Serikawa

**Affiliations:** 1 Institute of Laboratory Animals, Graduate School of Medicine, Kyoto University, Yoshidakonoe-cho, Sakyo-ku, Kyoto 606-8501, Japan

## Abstract

**Background:**

Hypohidrotic ectodermal dysplasia (HED) is a congenital disorder characterized by sparse hair, oligodontia, and inability to sweat. It is caused by mutations in any of three Eda pathway genes: ectodysplasin (*Eda*), Eda receptor (*Edar*), and Edar-associated death domain (*Edaradd*), which encode ligand, receptor, and intracellular adaptor molecule, respectively. The Eda signaling pathway activates NF-κB, which is central to ectodermal differentiation. Although the causative genes and the molecular pathway affecting HED have been identified, no curative treatment for HED has been established. Previously, we found a rat spontaneous mutation that caused defects in hair follicles and named it sparse-and-wavy (*swh*). Here, we have established the *swh *rat as the first rat model of HED and successfully identified the *swh *mutation.

**Results:**

The *swh*/*swh *rat showed sparse hair, abnormal morphology of teeth, and absence of sweat glands. The ectoderm-derived glands, meibomian, preputial, and tongue glands, were absent. We mapped the *swh *mutation to the most telomeric part of rat Chr 7 and found a Pro153Ser missense mutation in the *Edaradd *gene. This mutation was located in the death domain of EDARADD, which is crucial for signal transduction and resulted in failure to activate NF-κB.

**Conclusions:**

These findings suggest that *swh *is a loss-of-function mutation in the rat *Edaradd *and indicate that the *swh*/*swh *rat would be an excellent animal model of HED that could be used to investigate the pathological basis of the disease and the development of new therapies.

## Background

Hypohidrotic ectodermal dysplasia (HED) is a genetic disorder characterized by sparse hair, oligodontia, reduced sweating, and defects in a number of other ectodermal organs [1]. A lack of sweat glands can lead to recurrent severe overheating. Thus, children with HED are at substantial risk of sudden death in infancy due to fatal hyperpyrexia [2].

HED is caused by mutations in any of the three Eda pathway genes: ectodysplasin (*Eda*) [3,4], ED receptor (*Edar*) [5], and EDAR-associated death domain (*Edaradd*) [6]. They encode the ligand, receptor, and intracellular signal mediator of a single linear pathway, respectively. The Eda signaling pathway activates transcription factor NF-κB thereby playing an important role in embryonic development, especially in the development of ectodermally derived organs [1].

In humans, there are three types of HED with different inheritance: X-linked HED, autosomal dominant HED, and autosomal recessive HED. X-linked HED is the most common form of HED and is caused by mutations in *EDA*. Autosomal HED is caused by mutations in *EDAR *or *EDARADD*. Currently, over 100 different mutations in the *EDA *gene are known, while only ~20 and 4 causative mutations have been found in *EDAR *and *EDARADD*, respectively [7].

To date, four mouse models of HED are available: *Tabby, downless, Sleek*, and *crinkled*. The mutant phenotype of the *Tabby *mouse is inherited in an X-linked manner and the *Tabby *mouse carries a mutation in the *Eda *gene [4]. The recessive *downless *and dominant *Sleek *mice carry mutations in the *Edar *gene [8]. The *crinkled *mouse carries a mutation in the *Edaradd *gene [6]. The phenotypes in *Eda, Edar*, and *Edaradd *mutant mice are almost identical and include abnormalities in teeth, hair, and sweat glands, the triad of symptoms of HED. Over 20 different glands, including lacrimal, meibomian, salivary, submandibular, and mammary glands, are also affected [9-11]. These mutant mice have been used to study the roles of the Eda pathway in the development and morphogenesis of ectoderm-derived organs and to develop a novel treatment for HED using a recombinant EDA protein [12].

Mutations in some of the genes in the Eda pathway have been identified in various species, such as medaka [13], zebrafish [14], cattle [15-18], and dog [19]. Analyses of these mutations showed critical roles of the Eda pathway in the development of epithelial appendages, as well as in morphological evolution. Thus, the identification of novel mutations in different species emphasized the importance of the Eda pathway, and enabled the phenotypes of the mutated animals to be compared, giving new insights into the functions of the Eda pathway. If such novel mutations can be identified in mammals, then the affected species could be used as a disease model of HED.

In a previous study, we described a mutant rat, sparse and wavy hair (*swh*), which arose spontaneously in a colony of inbred WTC rats in 1998 [20]. The mutant phenotype is characterized by sparse and wavy hair, impaired body weight gain, and hypoplasticity of the mammary gland. The hair follicles in these rats were reduced both in number and size, a characteristic associated with hypoplasia of both the sebaceous glands and the subcutaneous fat tissues. The mammary glands of *swh/swh *female rats were hypoplastic and differentiation of mammary epithelial and myoepithelial cells was impaired. Thus, it is conceivable that the *swh/swh *rat will provide a good experimental model to clarify the mechanisms involved in the development of skin appendages, most of which are derived from ectoderm [20].

In our previously reported linkage analysis, *swh *mapped to the telomeric part of rat Chr 17. At that time, the physical location of the *swh *locus could not be accurately determined because a SSLP marker, *D17Rat140*, which defined the distal side of the *swh *locus was, in the earlier public rat genome linkage map, erroneously assigned to the middle part of Chr 17 and not to the telomeric part of Chr 17. Recently, with the development of more than 20,000 single nucleotide polymorphism (SNP) markers for 167 rat inbred strains and with the haplotype mapping data from the genotyping of these SNPs, the genome linkage map has been improved [21]. In the improved rat genome map, *D17Rat140 *and its neighboring genes are correctly mapped to the telomeric part of rat Chr 17. Thus, in addition to the 24 candidate genes selected from our previous linkage analysis, we also considered these newly mapped genes to be candidates of *swh *[20].

In this study, to demonstrate the suitability of the *swh *rat as an HED model, we investigated the pathology of tissues and organs in which morphological abnormalities in HED are known to occur. Furthermore, we identified the causative mutation of the *swh *phenotype using a positional cloning approach, and found a missense mutation in the death domain of EDARADD, that might explain the inability of the mutant *Edaradd *gene to activate NF-κB. Our findings suggest that *swh *is a loss-of-function mutation of the rat *Edaradd *and support the *swh/swh *rat as an excellent animal model of HED that can be used to investigate the pathological basis of the disease and to develop new therapies.

## Methods

### Animals

ACI/NKyo, WTC/Kyo, and WTC-*swh*/Kyo rats were provided by the Japanese National BioResource Project for the Rat and kept in our animal facility for all experiments in this study. Animal care and experimental procedures were approved by the Animal Research Committee, Kyoto University, Japan, and were conducted according to the Regulation on Animal Experimentation at Kyoto University.

### Histopathology

For light microscopy, the tongue, eyelid, ventral skin, footpad, and preputial gland were harvested from WTC-*swh/swh *and WTC rats at 8 weeks of age. Tissues were fixed in 10% neutral-buffered formalin, embedded in paraffin, and stained with hematoxylin and eosin (HE).

### Sweat tests and whole mount staining of mammary glands

The sweat test was performed as described previously [12]. Briefly, the hind paws of rats anesthetized with sevoflurane were painted with a solution of 3% (wt/vol) iodine in ethanol. Once dry, the paws were painted with a suspension of 40% (wt/vol) starch I mineral oil. Photographs were taken 1 min later and sweat was detected as dark spots. Mammary glands were prepared as a whole mount and stained as described previously [22].

### Fine mapping of swh

For fine mapping of *swh*, F2 animals (n = 769) were produced by intercrossing (ACI/NKyo × WTC-*swh*) F1 rats. Homozygous *swh/swh *animals were identified at 3-4 weeks of age based on the appearance of the sparse-and-waved hair phenotype. One hundred and ninety-eight *swh/swh *homozygotes were used for fine mapping of *swh*. Genomic DNA was prepared from tail biopsies using the automatic DNA purification system (PI-200; Kurabo, Japan).

### RNA extraction, RT-PCR and direct sequencing

Total RNA was extracted from the skin of 2-week-old animals. RNA preparation, RT-PCR and direct sequencing of PCR products were performed as described previously [23]. Rat *Edaradd *cDNAs were amplified with 6 sets of primers (Table 1). The PCR products overlapped each other and spanned the entire coding sequence of *Edaradd*.

**Table 1:** PCR primers used to amplify rat *Edaradd *cDNA

Primer set	Forward (5' > 3')	Reverse (5' > 3')
Edaradd-1&2	CTGAGAGAGAGTCGCGCATT	GCCACAGCTGTTCCCATAG
Edaradd-3&4	GCCCAGAAAAGGCAGCTC	GGAAAACCTTTGGAGTTTCTGA
Edaradd-5&6	CGATGAGCCAGCTTTACCTC	GGATAATTGGGTAACTATTCTCAACC
Edaradd-7&8	TCCATCCCAATTTTACCAACA	CGGCAAGCATTTTAATGACC
Edaradd-9&10	CAGTCAGCCCCTTGCACT	GCATGCTCTCATCAACATGG
Edaradd-11&12	TGTCACCAATGTGGTAGAAAAA	CAGGGATAACCACTGCCTGT

### Transient transfection and reporter assays

The NF-κB assay was designed to test for activation of the NF-κB responsive promoter. HEK293T cells grown in poly-L-lysine coated 24-well plates were transfected using SuperFect (Qiagen) with 1.2 μg pNF-κB-Luc (Clontech), 2 μg pRL-TK, and an increasing amount of expression vectors encoding the wild-type EDARADD or the *swh*-type EDARADD (Pro153Ser). The Luc reporter of the pNF-κB-Luc encodes firefly luciferase. The HSV-TK (herpes simplex virus thymidine kinase) promoter drives renilla luciferase in pRL-TK. Total DNA was adjusted to 2.6 μg by adding pCMV-HA (Clontech) vector as necessary. Luciferase activity was measured using the Dual-Luciferase Reporter Assay System (Promega) 48 h after transfection, according to the manufacturer's protocol.

## Results

### Phenotypes of *swh/swh *rat as hypohidrotic ectodermal dysplasia (HED)

**Figure 1:**
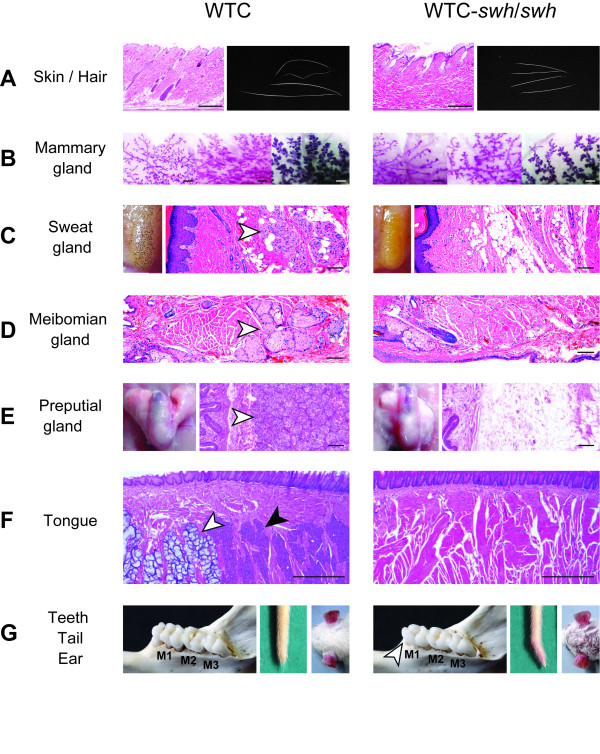
**Phenotypes of the swh/swh rat as hypohidrotic ectodermal dysplasia (HED)**. A, Sections of the dorsal skin (left) and hair (right). Incomplete hair follicles are evident in *swh/swh *rat. Scale bar, 0.5 mm. The WTC rat has four hair types; auchene, zigzag, awl and guard, while the *swh/swh *rat have only the abnormal awl hair. B, Whole mount stained mammary glands; 6-week-old (left), 8-week-old (center), and pregnant day 9 (right). Mammary gland branching is poor in *swh/swh *rat. Scale bar, 1 mm. C, Sweat test results (left) and section of the footpads. Sweat, detected as dark spots, is not seen in *swh/swh *rat. Sweat glands (arrowhead) are present in WTC rat and absent in *swh/swh *rat. Scale bar, 100 μm. D, Sections of the eyelid. The meibomian glands (arrowhead) are present in WTC rat and absent in *swh/swh *rat. Scale bar, 100 μm. E, An entire view (left) and a section of the preputial gland (right). The preputial gland is atrophied in male *swh/swh *rat. Acinous glands (arrowhead) are present in WTC rat and absent in *swh/swh *rat. Scale bar, 100 μm. F, Section of the tongue. Both mucous (open arrowhead) and serous (filled arrowhead) glands are present in WTC rat and neither is seen in *swh/swh *rat. Scale bar, 0.5 mm. **G**, Buccal views of lower molars (left), tip of tail (center), and posterior auricular region (right). Cusp number is reduced in the first molar (arrow head) in *swh/swh *rat. Some *swh/swh *rats show the kink tail. The bald patch behind the ear was not evident in the *swh/swh *rat.

Patients with HED display defective development of hair, teeth, sweat glands, and several exocrine glands, such as sebaceous, salivary, meibomian, and lacrimal [1,24]. To evaluate the relevance of the *swh/swh *rat as a HED model, we looked for developmental defects in those tissues of *swh/swh *rats. In addition to defects of the hair, skin, and mammary glands, which have been reported previously [20] (Figure 1A, B), we found defects in the sweat, meibomian, preputial, and tongue glands. In these tissues, the exocrine glands were absent in the *swh/swh *rats (Figure 1C, D, E, F). In the sweat test, no sweat was detected in *swh/swh *rats, indicating that the sweat glands were functionally defective (Figure 1C). We also found a reduced number of cusps in the lower first molars in the *swh/swh *rats (Figure 1G).

In the Eda pathway mutant mice, *Tabby, downless*, and *crinkled*, a kinked tail tip, a bald patch behind the ear, and abnormal pelage hair composition are characteristic. Similarly, in *swh/swh *rat, the pelage hair was composed of only an abnormal awl hair (Figure 1A); however, the tail had hair on it, the frequency of kinked tail was low, and the bald patch behind the ear was not found (Figure 1G).

These findings indicate that the mutant phenotypes of *swh/swh *rats are similar to developmental defects in HED patients and in the established mouse models; therefore, it is likely that the *swh/swh *rat will be suitable as a model of HED.

### Positional cloning of *swh*

**Figure 2:**
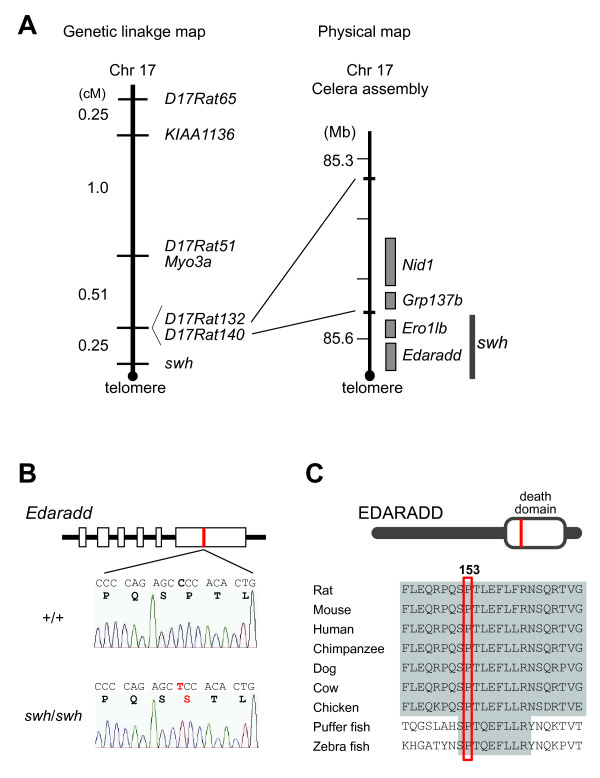
**Identification of the rat *swh *mutation**. A, Fine mapping of *swh *(left) and physical mapping of *swh *(right). The *swh *genetically mapped to the most telomeric part of rat Chr17, 0.25-cM distal from *D17Rat132 *and *D17Rat140*. In the physical map, the *swh *locus is localized to a ~0.2-Mb region between *D17Rat140 *and the telomere. Both *Ero1lb *and *Edaradd *have been mapped within the *swh *locus. B, Sequence analysis of *Edaradd *gene of wild-type and *swh/swh *rats. In the genomic DNA of *swh/swh *rat, a C to T (red) transition is present in exon 6 of rat *Edaradd *gene. This changes proline to serine at codon 153 of the deduced EDARADD protein. Rat codon 153 corresponds to codon 156 of mouse EDARADD isoform 1 (NP_598398) and codon 153 human EDARADD isoform B (NP_542776). C, Amino-acid sequence alignment of a region of the EDARADD death domain from different species. The 153rd amino acid that is altered in *swh/swh *rat is highly conserved in the vertebrates.

In a previous study, we mapped *swh *to rat Chr 17 [20]. To more specifically map the position of the *swh *locus, we genotyped F2 intercross progeny for markers known to be closely linked to *swh*. There was only one recombinant chromosome between *swh *and either *D17Rat132 *or *D17Rat140 *in 396 meioses (= 198 × 2) and we were able to map *swh *to the most distal part of Chr17 (Figure 2A). The rat genome map (RGSC v3.4) showed two genes in the *swh *locus, *Ero1lb *(ERO1-like beta (*S. cerevisiae*)) and *Edaradd *(ectodysplasin-A receptor-associated death domain). The mouse mutant of *Edaradd *is called *crinkled *(*cr*) and mice that carry this mutation show a sparse hair phenotype that is similar to that of the *swh *rat [25]. Additionally, mutations in the human *EDARADD *gene have been found in families affected with HED [6,26]. Thus, we considered *Edaradd *as a good candidate of *swh*. Although the abnormal expression of *Edaradd *mRNA was not detected in the skin of *swh/swh *rats (data not shown), we found a missense mutation (C to T) in exon 6 of the *swh/swh Edaradd *gene. This mutation was deduced to change proline to serine at the 153rd amino acid (Pro153Ser) of the rat EDARADD protein (Figure 2B). The 153rd amino acid is located in the death domain of EDARADD and is highly conserved in vertebrates (Figure 2C). These findings suggest that the Pro153Ser missense mutation of the *Edaradd *gene is causative of the phenotypes of *swh/swh *rats.

### Reporter assay for the Pro153Ser mutant EDARADD

**Figure 3:**
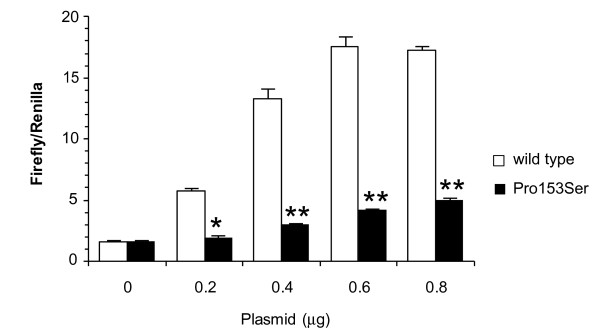
**Loss of NF-κB activation by the Pro153Ser Edaradd mutant protein**. 293T cells were transfected with 1.2 μg pNF-κB-luciferase reporter gene plasmid, 2 μg pRL-TK, and the amounts of each expression construct were measured in a reporter assay. The expression levels of luciferase were normalized to those of the internal control. Relative NF-κB activity in wild-type transfected cells increased in a dose-dependent manner, while significantly lower relative NF-κB activity was observed in the Pro153Ser *Edaradd*-transfected cells. *P < 0.01, **P < 0.001.

Overexpression of *Edaradd *in 293T cells activates NF-κB in a dose-dependent manner [25]. To examine whether Pro153Ser *Edaradd *can activate NF-κB, we carried out a reporter assay. As shown in Figure 3, wild-type *Edaradd *activated NF-κB in a dose-dependent manner. Meanwhile, Pro153Ser *Edaradd *showed significantly lower transcriptional activity of NF-κB than the wild type. The expression level of the Pro153Ser EDARADD protein detected by western blotting was not different from that of the wild type (data not shown). These findings indicate that the Pro153Ser missense mutation of the rat *Edaradd *gene could not activate NF-κB and that the Eda signaling pathway failed to function in *swh/swh *rats.

## Discussion

In this study, we demonstrated that the *swh/swh *rat harbored a Pro153Ser mutation in the *Edaradd *gene and showed typical symptoms of HED, such as sparse hair, oligodontia, inability to sweat, and developmental defects of the ectoderm-derived glands [27]. Hence, we successfully established the *swh/swh *rat as a genetically and phenotypically well-characterized disease model of HED.

EDARADD is a 208 amino acid protein consisting of an N-terminal Tnf receptor-associated factor (Traf)-binding consensus sequence and a C-terminal death domain (DD). The Traf-binding consensus sequence of EDARADD is used as a docking site for Traf1, Traf2, and Traf3, thereby recruiting Traf members and leading eventually to the activation of NF-κB [6]. The DD is involved in self-association of EDARADD and its interaction with EDAR [6,25]. Thus, EDARADD is central to Edar signaling. The N-terminal region is responsible for signal transduction and the C-terminal DD is required for receptor engagement.

To date, four *EDARADD *mutations have been found in a subset of human HED, one leads to autosomal dominant inheritance (Leu112Arg) [26], while the others lead to autosomal recessive inheritance (Glu142Lys, Pro121Ser, and Thr135-Val136del) [6,28,29]. All of these mutations are located in the DD and functional analyses showed that they resulted in the failure of EDARADD to interact with EDAR and to activate NF-κB. In the *crinkled *mouse, a genomic region of ~66-kb or more which includes exon 6 that encodes the entire DD, is deleted [25]. The *crinkled *mouse displays developmental defects in hair follicles, teeth, and sweat glands [30,31]. Hence, it is possible that a mutation in the DD of EDARADD is necessary for the HED syndrome to be manifested both in human and mouse.

All members of the DD superfamily form a highly compact structure comprising six antiparallel α-helix that is involved in homotypic and heterotypic protein-protein complex formation [32]. The region spanning the α1 to α4 helices of the DD of MyD88, a member of the death receptor superfamily, is required for its interaction with a downstream kinase [33]. A comparison of the amino acid sequences of the DD superfamily revealed that the Pro153Ser missense mutation found in the present study is located in the α4 helix of the DD of EDARADD. This mutation may cause a profound change in the polarity of a crucial region and eventually diminish NF-κB signaling. It is likely that Pro153Ser affects the structure of the DD thereby interfering in the interaction of EDARADD with EDAR.

Mutations affecting the Eda pathway are known in medaka [13], zebrafish [14], mouse [4,6,8], cattle [15-18], dog [19], and human [3,5,6]. Of them, the mouse mutants have been widely characterized as a model organism of HED. Here we report the *swh *mutation as the first example of a mutation in the Eda pathway in the rat.

Because the rat is closely related to the mouse, it is important to recognize how the rat *Edaradd *mutant phenotype matches the mouse Eda pathway mutant phenotypes. Similar to the mouse mutants, the *swh/swh *rat displayed sparse hair, misshapen teeth, and absence of sweating. Additionally, like the Eda pathway mutant, the *swh/swh *rat had only abnormal awl hair in the coat. The *swh/swh *rat showed a lack of the ectoderm-derived glands, meibomian, preputial, and tongue. Interestingly, both serous and mucous glands were absent in the tongue of the *swh/swh *rat. This is a clear difference from the mouse Eda pathway mutants that lacked mucous glands but had serous glands in the tongue [34]. Moreover, in contrast to the complete absence of tail hair in the Eda pathway mutant mice, the *swh/swh *rat had hair on its tail. The penetrance of the kink tail phenotype was low in the *swh/swh *rat, while almost all Eda pathway mutant mice showed the kink tail. Lastly, the bald patch behind the ear was not present in the *swh/swh *rat, although it was a very characteristic phenotype of the Eda pathway mutant mice.

Why these phenotypes are different between the Eda pathway mutant mice and the *swh/swh *rats is yet to be explained. However, different types of mutations could possibly explain the differences. The mouse *crinkled *mutation is a deletion [6], while the *swh *mutation is missense. Although the Luc-reporter assay strongly suggested that *swh *is a null mutation, the possibility that *swh *might be a hypomorphic mutation cannot be eliminated because the activation of NF-κB found in the assay was very low. In the Eda pathway mutant mice, the mammary, salivary and tracheal submucosal glands have been well characterized [9,10]. Further analyses of these glands in *swh/swh *rats will give further insights into the functions of the Eda pathway genes in the development of these glands.

## Conclusions

We successfully established the *swh/swh *rat as the first rat model of HED and identified *swh *as a Pro135Ser missense mutation in the *Edaradd *gene. The Pro135Ser mutant protein failed to activate NF-κB in the Eda signaling pathway. Thus, the *swh/swh *rat is a good model that can be used to investigate the pathological basis of HED.

## Authors' contributions

TK and MY performed the genetic and molecular biological experiments. RH and HH performed the histological examinations. TK wrote the paper and HH and TS revised the manuscript. All authors read and approved the final manuscript.
